# Hot Water in Sprains

**Published:** 1851-05

**Authors:** Samuel Jackson

**Affiliations:** Formerly of Northumberland


### Part 3.—Selections.

ARTICLE I.

Hot Water in Sprains.—By Samuel Jackson, M. D., formerly of Northumberland.

The immediate application of cold water in sprains is strongly recommended by M. Baudens, in a paper quoted at p. 235 of the preceding No. of this Journal. As my practice, at least for the last thirty-four years, has been the very opposite of this, and has yet led to equally desirable results, I beg leave to relate it on the present favorable occasion.

I was riding past the house of one of my patients thirty-four years ago and heard the screams of anguish: a woman had just sprained her ankle and was then suffering intensely. I ordered the foot to be put into water as hot as she could bear it and to be retained there until I should return—hot water to be added as the first became cool. In about an hour, I found that the pain had diminished almost from the very first minute, and that it was then entirely gone. She was put to bed with the foot greatly elevated, and after some hours, though there was no pain, towels dipped in cold water were freely applied and continued for several days. She was then perfectly well nor did she ever again suffer from that sprain.

Another strong case within my clear recollection is the following: A man sprained his ankle and suffered such severity of pain as to make him cry out most lustily. I was present in a few minutes and put his foot into hot water, immediately bleeding him largely from the arm as he sat in his chair bathing his foot. The pain became rapidly milder and I went into the next room to drink some tea. Looking over my shoulder after a few minutes, I saw his friends employed in fanning him and sprinkling his face with cold water. I ran to him, when to my horror, he was, as to human eyes, a mere corpse. I instantly tilted his chair, laying him flat on his back and ordered them to elevate his legs. Cold sprinkling and spirits of ammonia, now most fortunately in my pocket, were most diligently used, but it was an alarming time before he was restored. He was now put to bed entirely free from pain and the next day he pursued his journey in the stage without any inconvenience, having a flannel bandage applied.

This man told me he had no idiosyncrasy with respect to loosing blood; he was large, vigorous and healthy; hence the bleeding did not produce this alarming crisis. It was in a great measure the flaccidity of body and mind effected by a sudden transition from extreme suffering to perfect ease; though it must be apparent that the bleeding and bathing worked together so powerfully as to require more careful watching in any future case.

I once suffered a violent contusion of my elbow followed by intense pain: the arm was immediately put into a tub of hot water when it soon became entirely easy, requiring nothing further except rest. I have treated many other sprains and contusions in this way and I do not recollect a single case wherein the hot water failed of giving surprising relief.

I had been prepared for trying this method by reflections on the great comfort of warm bathing in many cases of conjunctivitis, before any considerable *error loci* had yet been formed; and on the fact, that in general relaxation of the system, there is less pain from parturition or any other violence.

How long that state of the part which is benefited by hot water, may generally continue after the accident, can hardly be defined. I have no recollection of using this remedy after a lapse of two hours, but I cannot be prepared of course to define the limits. If there has been time for inflammation to form, heat is inadmissible on my principle. Sometimes a tumour will instantly rise, but this being without inflammation, there can be no objection to the hot water.

It is very desirable to ascertain the best methods of refrigeration. M. Baudens keeps the foot night and day in a tub of cold water—a very inappropriate and inconvenient practice, if I am not greatly mistaken, for it prevents the proper position of the limb, which ought to be much elevated and evenly so from the acetabulum to the foot. Towels dipped in ice water and spread over the limb and bladders of snow or of pounded ice so placed that their weight may be supported by the pillows, and very conveniently applied. Ice or snow is particularly useful through the night when nurses and patients are sleepy and heat is sure to accumulate. A certain medium however must be observed with respect to the degree of cold, for it may easily be overdone unless the heat be great.

Suppose then a violent sprain has been relieved of all pain by hot water, let no one look upon the danger as past. The patient ought to be placed in bed with his foot greatly elevated, and after a few hours, cold ought to be applied even if the part is entirely easy. Inflammation may form, let us then prevent what every one knows is hard to cure in such parts. I have often seen lead water used and B. Bell has confidence in this and natural mineral waters, but truly I cannot believe they have any superiority over the pure fluid.

Low diet from the very first must be used in every case and purging too when the system will bear it; but if the patient is robust, he should lose blood from the arm. So much for the prevention of inflammation. I should not say a word about its cure had not M. Baudens advanced something bordering on novelty. He seems to have a horror of leeches because they may attract blood to the part. Now if the general arterial action has been lowered and the leg kept elevated, this horror need not be entertained. This we think would be the decision of a great majority of the profession in the present case. If I were called to a sprained ankle already in a state of severe inflammation I should certainly, after bleeding from the arm if necessary, apply an abundance of leeches and follow them up by cold, the limb being greatly elevated. B. Bell says, "No remedies I have ever employed answer so well as local bleeding:" and in the same page he further says, "When the injury has been severe we are obliged to apply leeches once and again. They require indeed to be repeated from time to time as long as any serious degree of pain continues."

After an indefinite time when all tendency to active spreading inflammation has been subdued and the little that is left is very feeble or confined to a small space, a very active large blister will generally absorb and carry it forthwith out of the body, but this is a perilous experiment and may do much harm if it do not fulfil our intention of extinguishing at once the whole disease, or of subduing it so far as to prevent reaction and thus to favor the operation of a second blistering. Whenever it has been determined to use this remedy, the part ought to be rubbed for fifteen minutes with *decoct. canthar. ex terebinth.* and an active plaster applied, so as to draw an effectual blister in the shortest time possible. The quick drawing of the blister is a point of the first importance in cases wherein you hope to absorb and carry off the whole disease. A slow blister is worse than none ; it is sure to irritate and increase the disease as sinapisms are known to do in similar cases. You are taken with pleurisy or peritonitis—some physicians would apply mustard with the hope of discussing a disease that is yet mild ; but *væ vobis*, you must loose more blood on account of the mustard and resort to a blister in the end. The best dressing by far for the first few days, is plantain or cabbage leaves ; but if the blister promise to run freely and not inflame, it may be soon dressed with mezereon or savin cerete, and if a copious discharge of pus be obtained, the disease will rapidly pass away. I can never forget the delighted countenance and applauding language of an old physician to whom I showed in my first year's practice, an ankle in this very condition. He had never known this use of savin, but from that day he used it freely and praised it highly. I had learned it from Crowther's work on white swellings.

Beware of warm poultices in the dressing of these blisters, for, as M. Baudens rightly says, "they favor in place of opposing the afflux of fluids to the part," and speaking of the long application of warm cataplasms, he says, "The long maceration the joint has been submitted to, deprives it of its elasticity, gives rise to a pasty engorgement and predisposes to the formation of white swelling." If it is determined not to use savin, the blister should be healed by the mildest dressings, so that another may soon be drawn ; thus the blistering may be conducted without any injurious irritation and made to absorb gradually and to carry off gently all the remaining inflammation. Dr. Rush used to talk and lecture much on his blistering point, and truly no idea or language can be more appropriate. The inflammation must be brought down to a low grade of action, or to a small periphery, so that a suitable blister will extinguish it at once, or so greatly diminish it that one or more subsequent blisters may be drawn with safety and success.

Of so much importance is it to guard against the irritation of blisters, that when I have applied them in the evening for critical diseases admitting of no delay, I have risen from my bed to bleed the patient if necessary at the time the plaster might begin to stimulate. When practicing in Northumberland, I have thus gone from one mile to four between midnight and morning to subdue the possible increase of fever either by the lancet or by additional doses of tartar emetic. By this means the evils of blistering may often be prevented; but as Hippocrates says, "the opportunity is fleeting:" if you wait till morning the pulse may be higher than it was in the evening and of course the blister has done much harm and no good. Sir John Pringle, in that early dawn of therapeutics, was better acquainted with this principle than many later authors in more enlightened times. *See* Part III. ch. ii. In the treatment of pleurisy when the bleeder was not present, he put on a blister and "was satisfied if the vein was opened before the flies had time to stimulate." I must observe however that his principle of practice was more commendable than the practice itself. More mischief can hardly be done by any remedy than by the drawing of a blister before the inflammation has been reduced to the blistering point. In Sir John's practice, the pain may have been scattered, but the inflammatory state remained and bleedings were then required which ought to have preceded the blister. It is very possible that when bleeding is inadmissible, nauseating doses of tart. emet. might be used to relax the system under the stimulation of a blister.

We have already entered our caveat against warm poultices in the dressing of blisters for sprains, and have approved M. Baudens' doctrine with respect to them; and lest any one should retort that our hot water may have the same bad effect, we must remind him, that we explode warmth after inflamation is formed. You may bathe a healthy limb in hot water for twenty-four hours and no engorgement will follow. I have bathed a great many sprained joints in the hottest water that could be borne without any of this evil. It is pain and inflammation that induce this engorgement, and these being both prevented by the hot bathing, this dreaded evil is prevented of course. But let this engorgement accrue and it will be greatly increased by much heat in any form. Yet there may be old cases in which hot water or steam may appear to revivify the torpid parts and render them sensible to curative means. But suppose you are called to an old case of this leuco-phlegmatic torpidity, is there a better remedy than frequent blistering that discharges freely? B. Bell recommends the pouring of warm bath or Buxton water on these engorged and torpid joints, but there is far more vivacity in the operation of cantharides, and the discharge not only carries off the evil stimulation, but it empties the vessels and promotes absorption.

Salivation is a last resort in certain protracted cases of sprains. I was called to a case wherein the metatarsal ligaments had been sprained twelve months before and the patient was now unable to walk on that limb. Rest, elevation of the leg, low diet, frequent cupping, and blistering were steadily pursued for nine months with much advantage; but there remained a painful state of the parts that prevented all use of them, and this without any evident swelling. Having reflected on the all-searching influence of mercury when parts supplied with infinitesimal vessels are inflamed as the iris, ligaments, and serous membranes, I determined to try its effects on the inflamed metatarsus. Calomel with blue pill was given and no sooner was the mouth sore than my patient felt with joy that his foot was greatly relieved. The change for the better was instantaneous and permanent. He was severely salivated but without any detriment; and I am glad to say that mercury in my hands has not since that time thirty-one years ago, transcended its just operation in a single case, and that I consider it an invaluable remedy not difficult to manage. It will cure chronic rheumatism, why not therefore a chronic sprain? But as I am not writing a treatise on sprains, I shall now return to my subject.

In nearly all cases of external violence which do not implicate any of the viscera, the immediate use of hot water is, as I sincerely believe, the best as it is the surest cure and preventive of pain. If you are about to have a tooth extracted, hold hot water in your mouth both before and after the operation: if you must have a felon lanced, hold the hand in hot water for a long time both before and after the cutting. My first case of what is vulgarly called "inverted toe nail" occurred to me after the patient had thoroughly relaxed the part by warm poulticing for many days, and I did not proceed to the operation of splitting the nail and eradicating the offending portion, till he had bathed his foot a long time in hot water. I had been taught in Dorsey's Surgery that it was a most painful operation, and I was therefore surprised, notwithstanding my hopes from the relaxation, to find the young man making very little complaint. I have several times performed this operation and owing as I believe to the hot bathing, I have not found it severe in a single case.

Now if I am not mistaken some reader will here exclaim, that even in inflammation, warm water agrees with some persons and cold with others. This fact however, I learned when a student from S. Cooper's prize essay on "Diseases of the Joints:" but however true this may be, I have not found a single case of bruise or strain in which hot water, when used in time, was not a great present comfort and permanent benefit.

When can I use the limb, is the continued cry of the patient and the continual anxiety of the harassed doctor. Some men have been known to walk off the gout, but this is a very dangerous experiment. I once sprained my metatarsus, but as the pain was not intolerable, I rode abroad without any application; on my return, I went to bed with the remittent fever and the pain was soon gone and forgotten. When I came to use the limb after two weeks, it became painful; but as the bilious fever prevailed greatly, I had no time to think of self, and nothing was done unless some rubbing with liniment. When the cold weather set in, the pain subsided gradually; but the warm weather of spring brought it back with distressing debility in the part. Thus going in the fall and coming in the summer, this infirmity continued to trouble me for four years. My limb was weak and painful every summer but not so bad as to send me to bed for a cure. I have seen many people triumph over the poor doctor by limping over the earth in great pain till nature cured the disease; but such wayward spirits always pay dearly for their folly, and they are sometimes finally brought to a bed of repentance.—*Amer. Jour. Med. Sci.*

